# Supplementary material for: Defining Elimination of Genital Warts—A Modified Delphi Study
Source: Vaccines (Basel). 2020 Jun 18;8(2):316. doi: 10.3390/vaccines8020316 (PMC7350253; doi:10.3390/vaccines8020316)
Supplement: Supplementary file 1 [file vaccines-08-00316-s001.pdf]

Supplementary Information Part 1:

Development of the proposed control and elimination thresholds for the Delphi Workshop

FACTORS TAKEN INTO CONSIDERATION IN DEVELOPING THE PROPOSED THRESHOLDS FOR PHASE-2 OF THE STUDY, THE DELPHI WORKSHOP:

In developing the proposed control and elimination thresholds, we considered some essential parameters around genital warts acquisition in heterosexual Australian residents, Australian men who have sex with men (MSM) and international travellers entering Australia. A brief account of these parameters is outlined below.

Populations:

Australian residents: We defined Australian residents as people who reside in Australia and have permission to remain permanently, either because they are: an Australian citizen, or the holder of a permanent visa. We further divided the Australian population into two sub-groups based on sexual orientation: i) heterosexual residents, and ii) MSM residents (including HIV positive MSM).

International travellers: We factored in importation of genital warts in Australia by incorporating cases related to international travellers. For our calculations, we considered sub-groups of international travellers, such as: i) backpackers, ii) newly commencing international students, iii) recently arriving immigrants and iv) recently arriving international female sex workers (FSW).

We defined 'recently arriving' as a traveller who has arrived within one year of their first visit to a clinic for a genital wart consult; and a 'new commencement' as a new international student enrolment in a particular course at a particular institution. For immigrants and international students that have been residing in Australia for more than one year, we assumed that they would most probably have genital warts prevalence similar to that observed in the Australian residents, and therefore are not included in our calculations.

Mathematical models for estimation of relative reduction in genital warts in each population:

We used three models for approximating relative reductions in the burden of genital warts in our three sub-sets of populations:

Heterosexual Australian residents: Model-based estimates of relative reduction in genital warts incidence in the heterosexual Australian population were calculated based on Korostil *et al* 2013 model.[1]

Australian MSM: Model-based estimates of relative reduction in incidence of HPV 6 and 11 in Australian MSM were calculated based on Zhang *et al* 2017 model.[2]

International travellers: Model-based estimates of relative reduction in prevalence of HPV 6 and 11 in international travellers were calculated based on Brisson *et al* 2016 pooled analysis.[3]

Population size:

Australian residents: For values of the baseline Australian population (heterosexual and MSM) and population projections for year 2021, 2030 and 2060, see Supplementary Table 1. Of note, for HIV-positive MSM, the population size and growth are based on the Australian HIV diagnosis and care cascade calculations.[4] We estimated rough population projections by assuming that the new diagnoses attributed to MSM is constant and equal to year 2016. We then assumed that the death rate, immigration rate and other calculation values stay the same as year 2016 values, and then projected them into the future. Based on these assumptions, we estimated the number of MSM living with diagnosed HIV (rounded to nearest 100) to be 12,000, 21,700, 28,000 and 45,700 for the years 2006, 2021, 2030 and 2060, respectively.

International travellers: For values of baseline population and projections of each sub-group of international travellers, see Supplementary Table 2.

Distribution of international travellers, by groups of home countries:

We assigned the international travellers entering Australia in two categories of countries (Supplementary tables 3–6): (a) from countries that have a quadrivalent HPV vaccination program (*q*HPV) (hereafter referred to as **group-1 countries**); and (b) from countries that either have a bivalent HPV vaccination program (*b*HPV), or no program (hereafter referred to as **group-2 countries**)

We assumed that these subgroups of international travellers were included in the estimated genital warts incidence at baseline (2006) in Australia,[5] and therefore, are not a part of the proposed relative reduction of genital warts incidence by Korostil *et al*,[1] which only included heterosexual Australian resident population. We thus expected that these subgroups will follow a different trajectory of reduction in genital warts incidence as compared to the heterosexual Australian residents depending on the vaccination coverage in the home country and their sexual mixing patterns in Australia.

Vaccination coverage and estimates of temporal relative reductions (RR)

(where information was not available for RR of genital warts incidence, RR of genital warts prevalence or HPV 6 and 11 prevalence or incidence estimates were used as proxies for RR of genital warts incidence)

Australian residents: For our calculations, the relative reduction in genital warts incidence in the heterosexual Australians was based on Korostil *et al* model's predictions[1] (Supplementary Table 1). Korostil *et al* predicted these reductions based on an assumption that 80% coverage for ongoing 2-dose vaccination in both sexes has been achieved. We applied the relative reduction in HPV 6 and 11 incidence in the Australian MSM population based on Zhang *et al* model's predictions[2] (Supplementary Table 1). Zhang *et al* predicted these reductions for an 84% coverage of 2 or more doses of boys' vaccination program. The real-time 2-dose vaccination coverage in boys (78%) and girls (85%) at the time of our calculations was comparable to the modelled assumptions.[6]

International travellers:

a) *Group-1 countries:* We calculated a weighted average of vaccination coverage for group-1 countries for each of the sub-groups, with weights being the proportion of travellers from each country (Supplementary Table 2 and Supplementary Tables 3–6). As all our sub-groups of international travellers attained an average vaccination

coverage of ≥50%, we assumed that group-1 countries in each sub-group of international travellers had achieved a 60% girls-only coverage. Also, we assumed that group-1 countries implemented their *q*HPV vaccination program in year 2012 (similar to the United Kingdom). We calculated the trajectory of relative reduction in genital warts prevalence for a 60% coverage presented in the Brisson *et al'*s pooled analysis.[3]

b) *Group-2 countries:* For group-2 countries, we assumed that some level of *q*HPV vaccination is offered through the private sector. Although there is no information available on what proportion of population gets vaccinated through this channel, we assumed a 5% *q*HPV girls-only vaccination coverage for our calculations that resulted in an estimated 4·0%, 5·5% and 7·5% relative reduction for year 2021, 2030 and 2060, respectively.

Baseline genital warts incidence:
(where information was not available for genital warts incidence, baseline genital warts prevalence or HPV 6 and 11 prevalence or incidence estimates were used as proxies for genital warts incidence)

Heterosexual Australian residents: Pirotta *et al* estimated an overall baseline incidence of genital warts of 21·9 per 10,000 population,[5] which was later on used in Korostil *et al* model to predict the relative reduction of genital warts incidence in the heterosexual Australian population.[1] However, Pirotta *et al* estimated incidence rate includes other sub-groups of populations in addition to the heterosexual population that seek treatment in general practice and sexual health clinics, such as the Australian MSM and international travellers. Therefore, we attempted to estimate the baseline incidence in the heterosexual population in Australia after subtracting the estimated new genital warts cases pertaining to other sub-groups from the total estimates of ~44,000 new cases at the baseline (see Methods). Subsequently, we calculated that for the heterosexual Australian population, the overall baseline incidence was 18·5 per 10,000 population.

Australian MSM: For the Australian HIV-negative MSM, we took the baseline anogenital warts incidence from the Health in Men (HIM) study.[7] Information is not available on genital warts incidence in HIV-positive Australian MSM. However, evidence suggests that seroprevalence of HPV 6/11/16/18 genotypes (combined) is significantly higher in HIV positive MSM at the anal canal and external genital sites as compared to HIV negative MSM.[8] This difference becomes insignificant when analysing HPV 6 and 11 separately. However, to make an estimate of genital warts incidence in Australian HIV-positive MSM, we extrapolated a difference of 1·9 times higher for genital and 1·5 times higher for anal warts in HIV-positive MSM as compared to the HIV-negative MSM.[8] This translated into the incidence rates of genital and anal warts in HIV positive MSM to be 1·81 and 2·89 per 100 person-years, respectively (overall 4·7 per 100 persons-year) which we used for our calculations.

International travellers: To ascertain the most appropriate age-specific baseline genital warts incidence in travellers, we first established which age group most international travellers belonged to in each sub-group (Supplementary Table 2). Genital warts incidence rates for these age groups were estimated based on the available information (Supplementary Tables 7–9). For international FSWs, baseline incidence was based on the incidence/prevalence of genital warts incidence in sex workers population in the home country, and not the age group most sex workers belong to (Supplementary Table 9). The majority of migrant FSWs in Australia are from the East Asian or Southeast Asian countries. Although there was no information available on the incidence of genital warts in the sex workers population in these countries, it was estimated that the prevalence of genital warts

in the FSWs is 23% in Thailand and 3·3% in China (Supplementary Table 10). For assumptions on values of baseline genital warts incidence for group-1 and -2 countries on FSWs see the notes on Supplementary Tables 2 and 10.

Sexual behaviour:

<u>Australian residents (heterosexual and MSM):</u> We did not consider sexual behaviour patterns for Australian residents. The reason being that the assumptions around condom use, efficacy of condom use, number of sexual partners and sexual mixing patterns were already modelled in the predictions proposed by Korostil *et al*[1] for the heterosexual population, and by Zhang *et al*[2] for the Australian MSM, the two models that we have used to estimate the trajectories of relative reductions in genital warts incidence in the Australian residents.

<u>International travellers:</u> To estimate the number of new genital warts cases in each sub-group of international travellers, it was important to consider certain factors around their sexual behaviour. For instance, what proportion of travellers have sex in Australia during their travels, and those who have sex, what proportion practice consistent condom use (Supplementary Table 2). Furthermore, it was crucial to understand their pattern of sexual mixing. Disassortative sexual mixing patterns where a low-risk person has sexual partnerships with high-risk persons, (in this case, an unvaccinated person from group-1 countries having a sexual encounter with a person from group-2 countries) will increase their risk of acquiring HPV infection and genital warts. Similarly, subgroups of internationals travellers from group-2 countries that maintain sexual contacts with people from their own countries with very low levels of sexual mixing with people from group-1 countries will show a less enhanced relative reduction in genital warts incidence. Information on sexual mixing patterns was only available for backpackers visiting Australia (Supplementary Table 2). For other sub-groups, no such evidence was available and therefore we made assumptions based on anecdotal feedback from sexual health and epidemiology experts at the Kirby Institute (Supplementary Table 2).

It was also important to consider the level of protection that consistent condom use offers against developing genital warts. Past research on people attending a sexual health clinic in Sydney showed that consistent condom use (always versus never) reduced the likelihood of genital warts development in men by 70% (crude OR 0·30, 95% CI: 0·20–0·40) and in women by 40% (crude OR 0·60, 95% CI: 0·40–0·90).[9,10] For our calculations, we took the two limits of the confidence intervals for both sexes (0·20 and 0·90) and took an average for these values (0·55). Therefore, we assumed that international travellers practicing consistent condom use will be 45% less likely to develop genital warts. For international FSWs, we assumed that those who practice consistent condom use will have a 40% less likelihood of genital warts development (Supplementary Table 2).

Supplementary Table 1: Values for parameters used in our calculations of the elimination-related thresholds for the Australian resident population (heterosexual and MSM)

Parameter	Values (source)	
	**Australian heterosexual residents**	**Australian men who have sex with men (HIV positive and negative combined)**
**Population size (n)**		
Baseline (2006) population size (Total Australian population: 20,091,504)[5]	19,901,504[a]	190,000 [7]
**Population projections [b]**		
	By year 2021: 25,849,184 [c]	By year 2021: 260,992 [2]
	By year 2030: 29,447,099 [c]	By year 2030: 301,073 [2]
	By year 2060: 40,219,029 [c]	By year 2060: 484,710 [2]
**Baseline genital warts incidence**		
	18·5 per 10,000 population[d]	470·9 per 10,000 population per year[e]
**Sexual behaviour**		
	.. [f]	.. [f]
**Modelled based assumption of *q*HPV vaccination coverage**		
	A coverage of 80% for two doses in both males and females[1]	A coverage of 84% boys' vaccination program of 2 or more doses[2]
**Modelled based approximations of relative reduction in new genital warts cases: [g]**		
- RR by year 2021	77·5% [1]	16% [2]
- RR by year 2030	90·5% [1]	45% [2]
- RR by year 2060	97%% [1]	99% [2]

RR: relative reduction

Notes:

[a] total population minus MSM population;

[b] total population projection by the Australian Bureau of Statistics (– CAT 3222.0: 2021: 26,110,176; 2030: 29,748,172; 2060: 40,703,739);[11]

[c] total population projections minus MSM population projections;

[d] see *Baseline Genital Warts Incidence for heterosexual Australian population*;

[e] see *Baseline Genital Warts Incidence in MSM population*;

[f] see *Sexual Behaviour in Australian residents*;

[g] based on modelled based relative reductions in genital warts incidence in heterosexual population[1] and relative reduction in the incidence of HPV 6 and 11 in MSM population[2]

Supplementary Table 2: Values for parameters used in our calculations of the elimination-related thresholds for international travellers (backpackers, newly commencing international student, newly arriving migrants and female sex workers)

Parameter	Values (source)			
	**Backpackers**	**Newly commencing international students (new commencements)** [a]	**Newly arriving immigrants** [b]	**Newly arriving international female sex workers**
**Population size** (n)				
- Baseline (2006)	548,695 [12]	184,276 [13]	236,094 [14]	Total Australian sex industry= 20,000 (mostly females);[15] 70% of the female sex workers are international,[15] and of these 30% arrive within one year of their first sexual health visit to a clinic.[16]
**Population projection** (average annual growth - %)				
	from baseline through to 2060: 1·6% [12]	from 2006-2021: 7·5% [13]	from baseline through to 2060: 1·6%[14]	from 2006-2021: 2·0% (assumption)
		from 2022-2030: 5·0% (assumption)		from 2022-2030: 1·5% (assumption)
		from 2031-2060: 3·0% (assumption)		from 2031-2060: 1·0% (assumption)
**Distribution of international travellers, by group of home countries based on the availability of HPV vaccination program** (%)				
- From group 1 countries	78% [12]	28% [13]	40% [14]	20% [15]
- From group 2 countries	22% [12]	72% [13]	60% [14]	80% [15]
**Age group with highest proportion of travellers** (%)				
	<24 years of age: >70%[17]	20–29 years of age: >71%[18]	20–39 years of age: ~60%[19]	25–34 years of age: ~55%[15]
**Baseline genital warts incidence, by age group**				
- For Group 1 countries	Group's average: 55·3 per 10, 000 young people aged 20–24 years	51 per 10, 000 people aged <30 years [20]	25·9 per 10, 000 people aged 20–40 years [20]	Group's average: 418 per 10, 000 sex workers
- For Group 2 countries	Group's average: 44·5 per 10, 000 young people aged 20–24 years	51 per 10, 000 people aged <30 years [20]	25·9 per 10, 000 people aged 20–40 years [20]	Group's average: 984·7 per 10, 000 sex workers
**Sexual behaviour**				
- Those who arrive without a long-term partner, that is, they are single (%)	80% [21]	..	..	..
- Of those who are single, proportion who have sex in Australia (%)	69% [21]	21% [22]	30% (assumption)	..
- Of those who have sex, proportion who practice consistent condom use (%)	50% [17]	50% [22]	50% (assumption)	95% [15]
- Of those who have sex, proportion who practice	50% [17]	50% [22]	50% (assumption)	5% [15]

inconsistent condom use (%)				
- Protection against contracting genital warts due to consistent condom use (% less likely) [c]	45% less likely to develop genital warts* [9]	45% less likely to develop genital warts [9]	45% less likely to develop genital warts* [9]	40% less likely to develop genital warts* [9]
- Proportions who have sex with travellers from their own country [d] (%)	31% [17]	70% (assumption) [e]	30% (assumption) [e]	..
- Proportions who have sex with travellers from a different country [d] (%)	46% [17]	20% (assumption) [e]	20% (assumption) [e]	..
- Proportions who have sex with a local resident, that is with an Australian resident (%)	23% [17]	10% (assumption) [e]	50% (assumption) [e]	..
***q*HPV vaccination coverage in groups of countries**				
- Group 1 countries	60%[α]	60% [α]	60% [α]	60% [α]
- Group 2 countries	5% (assumption) [α]	5% (assumption) [α]	5% (assumption) [α]	5% (assumption) [α]
**Modelled based relative reductions (RR) in prevalence of HPV 6 & 11 [f]**				
**Group-1 countries**				
- RR by year 2021	33%	33%	33%	33%
- RR by year 2030	77%	77%	77%	77%
- RR by year 2060	84%	84%	84%	84%
**Group-2 countries**				
- RR by year 2021	4·0% (assumption) [e]	4·0% (assumption) [e]	4·0% (assumption) [e]	4·0% (assumption) [e]
- RR by year 2030	5·5% (assumption) [e]	5·5% (assumption) [e]	5·5% (assumption) [e]	5·5% (assumption) [e]
- RR by year 2060	7·5% (assumption) [e]	7·5% (assumption) [e]	7·5% (assumption) [e]	7·5% (assumption) [e]

RR: relative reduction

Notes:

[a] for our calculations, we included international students' commencements in Higher Education (HE), Vocational Education and Training (VET), English Language Intensive Courses for Overseas Students (ELICOS), and non-reward courses, and excluded international students studying in schools.[13]

[b] for our calculations, we included immigrants that entered Australia on off-shore permanent visa, onshore/offshore temporary visa, humanitarian visa, and New Zealanders permanently settling in Australia annually. We excluded on-shore permanent applicants as these applicants are either temporary residents or international students which we already included in our calculations. We also excluded residents entering on family stream visa which includes children, partners and parents of immigrants.

[c] see *Sexual Behaviour (international travellers)*

[d] we assumed that international travellers who have sexual partners from 'own country' or a 'different country' will each have the same distribution of proportions from group 1 and group 2 countries as shown in the table above. For instance, for backpackers, 31% and 46% have sexual partners from their own country or a different country, respectively, and each of these proportions were further stratified into: 78% have sex with travellers from group 1 countries, and 22% have sex with travellers from group 2 countries (see equation in Supplementary Table 11)

[e] assumptions were made based on feedback from sexual health and epidemiology experts at the Kirby Institute

[f] see *Vaccination coverage and estimates of temporal relative reductions*

Supplementary Table 3: Percentage distribution of backpackers visiting Australia, by home country and availability of qHPV vaccination program

Countries	Average proportion of backpackers arriving in Australia (%), 2006–2015 [12]	≥2-dose *q*HPV vaccination coverage (%) in age-eligible groups
**Group-1 countries**		
UK	19·9%	Girls 85% [23]
Germany	10·8%	Girls 40% [24]
USA	7·8%	Girls and boys 43% [25]
France	6·0%	Girls 17% [26]
NZ	5·8%	Girls 66% [27]
Scandinavia	5·1%	Girls 80% [28-30]
S. Korea [a]	5·4%	Girls 42%
Canada	4·1%	Girls and boys 56% [31]
Switzerland [b]	2·6%	Girls ~47% [32]
Italy	2·2%	Girls ~70% [33, 34]
Malaysia	2·1%	Girls 99% [35]
Singapore	1·7%	Girls 04% [36]
‘Other’ Europe [c]	1·6%	Girls 68%
‘Other’ countries [d]	3·2%	Girls 64%
**Group-2 countries**		
Japan	4·9%	..
China/Hong Kong	3·5%	..
Taiwan	2·6%	..
Netherlands	2·6%	..
‘Other’ Europe	6·2%	..
‘Other’ Countries	1·8%	..

78% are from countries with a *q*HPV program –

Group’s weighted average of vaccination coverage= 59%

22% are from countries with a *b*HPV or no program

Notes:
[a] unpublished data – Korean CDC (KCDC. NIP (2-,4-valent HPV vaccines) coverage rate (as of 7. Aug 2017) – courtesy Seqirus
[b] varies by regions – median of regions taken
[c] three out of 14 ‘other’ European countries have a qHPV vaccination program in place, namely Slovenia, Spain, and Ireland with 55%,[37] 77%[38] and 72%[39] vaccination coverage, respectively; generating an average of 68% coverage for ‘other’ Europe.
[d] seven out of 11 ‘other’ countries have a qHPV vaccination program, in place, namely Israel, Peru, Portugal, San Marina, Uruguay, Argentina, and Chile. Vaccination coverage data is available on Israel (10%),[40] Portugal (88%),[41] Argentina (71%)[42] and Chile (85%)[43]; generating an estimated average of 64% coverage for ‘other’ countries

Supplementary Table 4: Percentage distribution of newly commencing international students in Australia, by home country and availability of qHPV vaccination program

Countries	Average proportion of international students arriving in Australia (%) 2006–2015 [13]	≥2-dose qHPV vaccination coverage (%) in age eligible groups
**Group-1 countries**		
USA	3·0%	Girls and boys 43% [25]
Malaysia	3·0%	Girls 99% [35]
Brazil [a]	4·0%	Girls 50%
S. Korea [b]	5·7%	Girls 42%
Other [c]	13·0%	50%
**Group-2 countries**		
China	24·0%	..
India	13·0%	..
Japan	2·6%	..
Nepal	3·0%	..
Thailand	4·6%	..
Vietnam	4·0%	..
Indonesia	3·0%	..
Other	17·4%	..

28% are from countries with a *q*HPV program

Group’s weighted average of vaccination coverage= 53%

72% are from countries with a *b*HPV or no program [d]

Notes:

[a] unpublished vaccination coverage data, Ministry of Health, Brazil, courtesy Seqirus

[b] unpublished vaccination coverage data – Korean CDC (KCDC. NIP (2-,4-valent HPV vaccines) coverage rate (as of Aug 2017) – courtesy Seqirus

[c] there are 39 countries in the 'other' category of group-1 countries. Vaccination coverage details are available for Canada (56%),[31] Chile (85%),[43] Colombia (87%),[44] France (17%),[26] Germany (40%),[24] Ireland (72%),[39] Italy (95%),[33,34] Norway,(76%)[29] Singapore (4%),[36] Sweden (77%),[45] Switzerland (47%),[32] Portugal (88%),[41] and United Kingdom (85%),[23] generating a weighted average of vaccination coverage of 50% for the 'other' group-1 countries.

[d] of note, in this group, around 14% of newly arriving international students are from countries with a pilot *q*HPV program in place (Thailand, Vietnam & Indonesia)

Supplementary Table 5: Percentage distribution of newly arriving immigrants, by home country and availability of qHPV vaccination program

Countries	Proportion of the total newly arriving immigrants, 2006–2015 [46]	2-dose *q*HPV vaccination coverage (%) in age eligible groups
**Group-1 countries**		
UK	12·7%	Girls 85% [23]
NZ	9·9%	Girls 66% [27]
Ireland	3·8%	Girls 72% [39]
USA	2·0%	Girls and boys 43% [25]
Malaysia	1·4%	Girls 99% [35]
Canada	0·7%	Girls and boys 56% [31]
France	0·7%	Girls 17% [26]
Other OCED [a]	5·1%	68%
other countries with *q*HPV [b]	3·2%	··
**Group-2 countries**		
India	17·6%	··
China	9·6%	··
Philippines	5·3%	··
S. Africa	1·6%	··
Vietnam	1·5%	··
Nepal	1·4%	··
Other OCED	5·5%	··
Other	17·9%	··

Group-1: 40% are from countries with a *q*HPV program. Group's weighted average of vaccination coverage= 81%

Group-2: 60% are from countries with a *b*HPV or no program

Notes:

[a] There are 35 Organisation for Economic Co-operation and Development (OECD) countries. Six are already shown in the list of group-1 countries, namely Canada, France, Ireland, New Zealand, United Kingdom and United States. 14 out of the remaining 29 OCED countries have a national *q*HPV program, namely Austria, Chile (85%),[43] Czech Republic, Germany (40%),[24] Israel(10%),[40] Italy (95%),[33, 34] South Korea (42%) (see table 3 notes), Norway (76%),[29] Portugal (88%),[41] Slovenia (55%),[37] Spain (77%),[38] Sweden (77%),[45] and Switzerland (47%),[32] generating a group's average of 68% of coverage (proportional distribution of individual countries in this category was not available hence a weighted average could not be calculated).

[b] other countries with *q*HPV' - 30 countries other than the European / Western countries have a *q*HPV vaccination program in place, and information is not available on most of their vaccination coverage (for our calculations, we have assumed a 40% coverage for these group of countries).

Supplementary Table 6: Percentage distribution of newly arriving international female sex workers in Australia, by home country and availability of *q*HPV vaccination program

Countries	Proportion of the total newly arriving female sex workers in Australia[15]	2-dose *q*HPV vaccination coverage (%) in age eligible groups
S. Korea [a]	9·0%	Girls 42%
New Zealand	5·0%	Girls 66% [27]
Other [b]	5·7%	61%
Thailand *(pilot qHPV)*	44·0%	..
China *(no HPV prog)*	26·0%	..
Other	10·3%	..

20% are from countries with a *q*HPV program. Group's weighted average of vaccination coverage= 54%

80% are from countries with a *b*HPV or no program

Notes:

[a] unpublished data – Korean CDC (KCDC. NIP (2-,4-valent HPV vaccines) coverage rate (as of 7. Aug 2017) – courtesy Seqirus

[b] 10 out of 28 'other' countries have a *q*HPV vaccination program in place, namely (where data is available vaccination coverage is shown within brackets): Brazil (37%),[47] England (85%),[23] Fiji Island, Ireland, (72%)[39] Germany (40%),[24] Malaysia (99%),[35] Scotland (80%),[48] Spain (77%),[38] Sweden (77%),[45] and USA (43%);[25] generating an estimated average of 61% coverage for this group of countries (proportional distribution of individual countries in this category was not available hence a weighted average could not be calculated).

Supplementary Table 7: Pre-vaccination incidence rates of genital warts in young people aged 20-24 years, by country

			Incidence per 10,000	
**Countries**	**Study**	**Study period**	**Overall**	**20–24 years**
**a. Group-1 Countries**				
United Kingdom	Desai *et al*, 2006[49]	2006–2008	15·7	75·5
Germany [a]	Kraut *et al*, 2010[50]	2004–2006	17·0	48·9
USA	Camenga *et al*, 2013[51]	2000–2005	23·0	47·0
France	Monsonégo *et al*, 2007[52]	2005	22·9	50·0
New Zealand	…	..	..	..
**Group's average incidence**			**19·6**	**55·3**
**b. Group-2 Countries**				
China (Hong Kong)	Lin *et al*, 2010[53]	2009	20·4	..
Netherlands [b]	van den Broek *et al*, 2010[54]	2006	11·6	..
Japan [c]	Sasagawa *et al*, 2011[55]	2009	25·1	..
Taiwan [a]	Hsueh P-R, 2009 (overall);[56]	2003; 2000-2013	6·2	13·0
	Cho C-Y *et al* 2017 (20-24 years)[57]			
**Group's average incidence**			**15·8**	**~44·5 [d]**

Notes:

[a] taking average between sexes for 20–24 years age group

[b] taking average between sexes (based on any case: new and recurrent)

[c] showing genital warts incidence for females only, for males the incidence is unknow

[d] if in group-1 countries, the average genital warts incidence in young people is about three-fold the overall average, then it could be assumed that the same is true for the group-2 countries, giving an estimated incidence of 44·5 per 10,000 population in young people (aged 20–24 years)

Supplementary Table 8: Pre-vaccination incidence rates of genital warts in people aged <30 years, by sex

Study		New genital warts incidence per 10,000 population, by sex		Average incidence per 10,000 population, by sex
	Age group	Range		
Buckley *et al*, 2016[20]	men <30 of age	13·0	56·0	34·5
	women <30 of age	32·0	103·0	67·5
	**Average between sexes**			**51·0**

Supplementary Table 9: Pre-vaccination incidence rates of genital warts in people aged 20-40 years, by sex

Study		New genital warts incidence per 10,000 population, by sex		Average incidence per 10,000 population, by sex
	Age group	Range		
Buckley *et al*, 2016[20]	men 20–40 years of age	7·7	45·3	26·5
	women 20–40 years of age	7·6	43·0	25·3
	**Average between sexes**			**25·9**

Supplementary Table 10: International female sex workers: Prevalence of genital warts in female sex worker population in home country

Countries	Study	Study period	Prevalence of genital warts in female sex workers
**Group 1 countries** *a*			
S. Korea	..	..	unknown
New Zealand	..	..	unknown
Other	..	..	..
**Group 2 countries** b			
Thailand *(pilot qHPV)*	Leaungwutiwong *et al, 2015*[58]	study period is not specified	23·0%
China *(no HPV prog)*	Su *et al, 2015*[59]	2000–2011	3·27%
Other			..

Notes:
a Group's average of 418 per 10, 000 sex workers (see Supplementary Table 2) taken as the baseline incidence of genital warts – workings of estimating the group's average are: we assumed S. Korea will have a prevalence similar to China's baseline genital warts prevalence in female sex workers of 327/10,000 sex workers; we assumed New Zealand will have a prevalence similar to Australia's baseline genital warts prevalence in FSWs prevalence of 600/10,000 sex workers)[60]; we assumed that 'other' group of countries has a prevalence similar to China's baseline genital warts prevalence in female sex workers of 327/10,000 sex workers)[59]
b Group's average of 984·7 per 10, 000 sex workers (see supplementary table 2) taken as the baseline incidence of genital warts – workings of estimating the group's average are: Baseline genital warts prevalence in female sex workers in Thailand of 2300/10,000 sex workers); Baseline genital warts prevalence in FSWs in China of 327/10,000 sex workers); 18 out of 28 countries in 'other' category have no *q*HPV vaccination program in place. We assumed that this group of countries has a prevalence similar to China's baseline genital warts prevalence in FSWs of 327/10,000 sex workers)^
(..) no data available

Methods:

We calculated new genital warts cases for each sub-group of populations separately for our time points - years 2006, 2021, 2030 and 2060. We first calculated the number of new cases in Australian MSM and international travellers at baseline (details given below), and then deducted these from the total of 43,937 new cases estimated by Pirotta *et al* in the pre-vaccination era. By doing so we estimated the number of new cases in the heterosexual Australian population at baseline, and thus calculated a revised proportion of 'new' cases in Pirotta *et al*'s sample that was attributable to heterosexual Australians only

(an important factor in estimating the number of new cases in the heterosexual population for our future time points).

We estimated the number of new cases related to Australian MSM at baseline by taking the incidence of anogenital warts (AGW) of 2·86 per 100 person-years.(42) Based on our assumptions of 1·6% annual growth in the MSM population, we then estimated the number of new AGW cases that would have been observed in this sub-group over our timeline in the absence of a vaccination program. We then applied the relative reductions of incidence of HPV subtype 6 and 11 as predicted by Zhang *et al*[2] and estimated the number of new cases of AGW in Australian MSM through to year 2060.

We then calculated the number of new cases that would have been expected in heterosexual Australians for years 2021, 2030 and 2060 in the absence of a vaccination program. We did this by: Firstly, multiplying the per capita general practice (GP) consults of 4·72 for the year 2016 (**http://medicarestatistics.humanservices.gov.au/statistics/mbs_group.jsp**) with the estimates of the Australian population projections by the Australian Bureau of Statistics – CAT 3222·0(6) for years 2021, 2030 and 2060 to get an estimated total annual GP consults in Australia for each time point. And secondly, by extrapolating the revised Pirotta *et al*'s[5] sample estimates of new genital warts GP consults in heterosexual residents to our estimated national annual GP consults for all time points. The number of new consults were then multiplied by 1.298 to account for genital warts cases managed in sexual health clinics. We then applied the relative reductions predicted by Korostil *et al*[1] over three time-points - 2021, 2030 and 2060 to these estimates.

For all sub-groups of international travellers, we first factored in our assumptions around sub-groups' population size, age-specific baseline incidence of genital warts, and sexual behaviour to estimate the number of cases relating to each of these groups at baseline (see Supplementary Table 2). Subsequently, we applied the relative reduction trajectory for 60% vaccination coverage as per the Brisson *et al*[3] pooled analysis predictions for group-1 countries, and the assumed relative reduction trajectory of 5% vaccination coverage for group-2 countries over three time-points – 2021, 2030 and 2060. Details of stratification and equations used for our calculations for subgroups of international travellers is shown in Supplementary Table 11 and in section 'additional equations' (with backpackers' subgroup as an example). For travellers having sex with Australian residents, we calculated the number of new cases by taking Australia's overall incidence at the baseline and applying the relative reductions as per Korostil *et al*[1] model's predictions of 77.5%, 90.5% and 97% for 2021, 2030 and 2060, respectively.

Data analysis:

The number of genital warts cases in Australian residents (heterosexual and MSM) and international travellers for all time-points were calculated in Microsoft Excel 2016 software (Supplementary Table 11 for formulae), and their binomial exact 95% confidence intervals (95% CI) were calculated using

Stata version 14·1. Point estimates and their binomial exact 95% CI for relative reductions of genital warts incidence were calculated using Stata version 14·1.

Supplementary Table 11: Equations for calculations of new genital warts cases in the sub-groups of international travellers, for each time point (example Backpackers' sub-group)

Strata	Notation	Equation
Number of sexually active backpackers in Australia at a given time point t*i*, (where *i*=2006, 2021, 2030 and	a_{ti}	
2060)		
50% backpackers practice inconsistent condom	b_{ic}	$b_{ic} = a_{ti} * 0{\cdot}50$
- 31% have sex with travellers from their own country (31% of b_{ic})	x_{ic}	$x_{ic} = b_{ic} * 0{\cdot}31$
- 78% have sex with travellers from group-1 countries - number of genital warts cases in this sub-group	x_1	$x_1 = (x_{ic} * 0{\cdot}78) * Ig_1t_{i\ /10{,}000}$
(taking group-1 countries' genital warts incidence at a given time point= Ig_1t_i) [a]		
- 22% have sex with travellers from group-2 countries - number of genital warts cases in this sub-group	x_2	$x_2 = (x_{ic} * 0{\cdot}22) * Ig_2t_{i\ /10{,}000}$
(taking group-2 countries' genital warts incidence at a given time point= Ig_2t_i)		
- 46% have sex with travellers from other countries (46% of b_{ic})	y_{ic}	$y_{ic} = b_{ic} * 0{\cdot}46$
- 78% have sex with travellers from group-1 countries - number of genital warts cases in this sub-group	y_1	$y_1 = (y_{ic} * 0{\cdot}78) * Ig_1t_{i\ /10{,}000}$
(taking group-1 countries' genital warts incidence at a given time point= Ig_1t_i) [a]		
- 22% have sex with travellers from group-2 countries - number of genital warts cases in this sub-group	y_2	$y_2 = (y_{ic} * 0{\cdot}22) * Ig_2t_{i\ /10{,}000}$
(taking group-2 countries' genital warts incidence at a given time point= Ig_2t_i) [a]		
- 23% have sex with Australian residents – number of genital warts incidence in this sub-group: (23% of b_{ic})	z_{ic}	$z_{ic} = b_{ic} * 0{\cdot}23 * Iat_{i\ /\ 10{,}000}$
(taking Australia's genital warts incidence at a given time point= I_at_i) [a]		
50% backpackers practice consistent condom (and therefore are 45% less likely to develop genital warts)	b_c	$b_c = a_{ti} * 0{\cdot}50$
- 31% have sex with travellers from their own country (31% of b_c)	x_c	$x_c = b_c * 0{\cdot}31$
- 78% have sex with travellers from group-1 countries - number of genital warts cases in this sub-group	x_3	$x_3 = x_1 – (x_1 * 0{\cdot}45)$
(taking group-1 countries' genital warts incidence at a given time point= Ig_1t_i)		
- 22% have sex with travellers from group-2 countries - number of genital warts cases in this sub-group	x_4	$x_4 = x_2 – (x_2 * 0{\cdot}45)$
(taking group-2 countries' genital warts incidence at a given time point= Ig_2t_i)		
- 46% have sex with travellers from other countries (46% of b_c)	y_c	$y_c = b_c * 0{\cdot}46$
- 78% have sex with travellers from group-1 countries - number of genital warts cases in this sub-group	y_3	$y_3 = y_1 – (y_1 * 0{\cdot}45)$
(taking group-1 countries' genital warts incidence at a given time point= Ig_1t_i)		
- 22% have sex with travellers from group-2 countries - number of genital warts cases in this sub-group	y_4	$y_4 = y_2 – (y_2 * 0{\cdot}45)$
(taking group-2 countries' genital warts incidence at a given time point= Ig_2t_i)		
- 23% have sex with Australian residents – number of genital warts incidence in this sub-group: (23% of b_c)	z_c	$z_c = z_{ic} – (z_{ic} * 0{\cdot}45)$
(taking Australia's genital warts incidence at a given time point= I_at_i)		

Notes:

[a] Ig_1t_i, where Ig_1= incidence in group-1 countries, and t_i = a given time point - 2006, 2021, 2030 and 2060; Ig_2t_i, where Ig_2= incidence in group-2 countries, and t_i = a given time point - 2006, 2021, 2030 and 2060; I_at_i, where I_a incidence in Australia, and t_i = a given time point, 2006, 2021, 2030 and 2060

Of note, modelled relative reductions were applied to genital warts' incidences for each country type (group-1, -2 and Australia) and for each subset of international travellers for timepoints 2021, 2030 and 2060

Additional equations (example backpackers' sub-group):

Number of new genital warts cases in backpackers who practice inconsistent condom use at a given time point: $A_i = x1 + x2 + y1 + y2 + z_{ic}$

(where *i* = 2006 (baseline), 2021, 2030 and 2060); *ic* = inconsistent condom use

Number of new genital warts cases in backpackers who practice consistent condom use at a given time point: $B_i = x3 + x4 + y3 + y4 + z_c$

(where *i* = 2006 (baseline), 2021, 2030 and 2060); *c* = consistent condom use

Total number of new genital warts cases in backpackers at a given time point = $A_i + B_i$

Supplementary Figure 1: A flow chart of steps of estimating the consolidated number of new genital warts cases in Australia (Australian residents plus international travellers) for each time point*

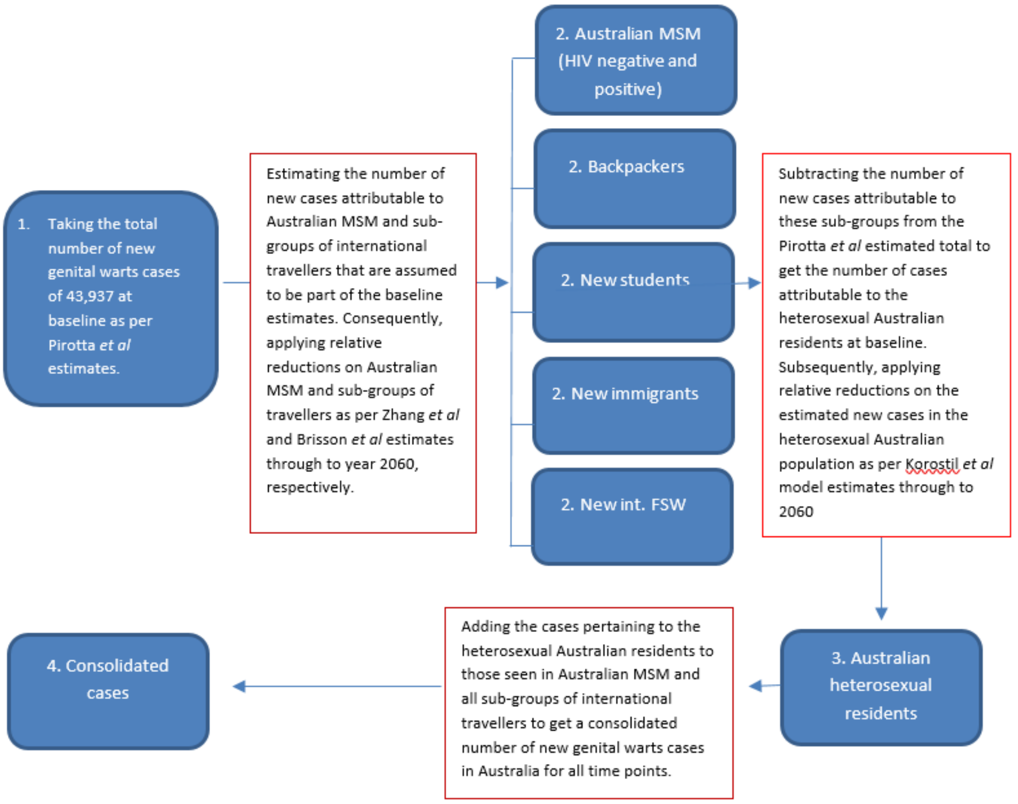

*MSM: men who have sex with men; Int. FSW: international female sex workers.

Results:

Supplementary Table 12: Estimates presented at the Delphi workshop for number and relative reduction in new genital warts consults in Australia, by sub-population type

	Australian heterosexual population*	Total number[3] of 'new' genital warts/year (95% CI)	New genital warts rates per 10,000 population (95% CI)	Relative reduction (%) (95% CI)
**Baseline (2006)**	19,901,504	36,719 (31,579–42,181)	18·4 (15·9–21·2)	
**2021***	25,849,184	10,419 (9,849–13,155)	4·0 (3·8–5·1)	78·2 (77·7–78·6)
**2030**	29,447,099	5,012 (4,350–5,811)	1·7 (1·5–2·0)	90·8 (90·5–91·0)
**2060**	40,219,029	2,162 (1,876–2,506)	0·5 (0·5–0·6)	97·2 (97·1–97·3)
	**Australian MSM population**	**Total number of 'new' anogenital warts/year (95% CI)**	**New anogenital warts rates per 10,000 Australian MSM (95% CI)**	**Relative reduction (%) (95% CI)**
**Baseline (2006)**	190,000	5,656 (5,512–5,803)	297·7 (290·1–305·4)	
**Year 2021**	260,992	6,593 (6,437–6,752)	252·6 (246·6–258·7)	15·1 (12·1–18·1)
**Year 2030**	301,073	5,034 (4,897–5,174)	167·2 (162·7–171·8)	43·8 (41·7–45·9)
**Year 2060**	484,710	216 (188–247)	4·5 (3·9–5·1)	98·5 (98·3–98·7)
	**Backpackers arriving each year**	**Total number of 'new' genital warts/year (95% CI)**	**New genital warts rates per 10,000 backpackers (95% CI)**	**Relative reduction (%) (95% CI)**
**Baseline (2006)**	548,695	1,075 (1012–1141)	19·6 (18·4–20·8)	
**2021**	696,204	911 (849–972)	13·1 (12·3–14·0)	33·2 (27·1–38·8)

**2030**	803,119	525 (481–572)	6·5 (6·0–7·1)	66·6(63·0–69·9)
**2060**	1,292,978	699 (654–759)	5·4 (5·1–5·9)	72·2 (69·4–74·7)
	**New commencements-**	**Total number of 'new' genital**	**New genital warts rates per**	**Relative reduction (%)**
	**International students**	**warts/year (95% CI)**	**10,000 new int. students (95% CI)**	**(95% CI)**
**Baseline (2006)**	184,276	144 (121–170)	7·8 (6·5–9·2)	
**2021**	545,250	362 (326–401)	6·6 (5·9–7·3)	15·0 (-3·1–30·0)
**2030**	845,862	474 (432–519)	5·6 (5·1–6·1)	28·3(13·6–40·5)
**2060**	2,053,129	1,095 (1032–1163)	5·3 (5·0–5·6)	31·7 (18·7–42·6)
	**Newly arriving**	**Total number of 'new' genital**	**New genital warts rates per**	**Relative reduction (%)**
	**immigrants**	**warts/year (95% CI)**	**10,000 new immigrants (95% CI)**	**(95% CI)**
**Baseline (2006)**	236,094	119 (99–142)	5·0 (4·1–6·0)	
**2021**	299,565	85 (68–105)	2·8 (2·2–3·5)	43·7 (25·6–57·4)
**2030**	345,568	70 (55–88)	2·0 (1·5–2·5)	59·8 (46·0–70·1)
**2060**	556,346	99 (82–123)	1·7 (1·4–2·2)	64·0 (53·0–72·4)
	**Newly arriving**	**Total number of 'new'**	**New genital warts rates per**	**Relative reduction (%)**
	**international**	**genital warts/year (95% CI)**	**10,000 newly arriving FSWs (95% CI)**	**(95% CI)**
	**female sex workers**			
**Baseline (2006)**	4,060	224 (196–254)	552·2 (483·4–626·4)	
**2021**	5,464	276 (245–310)	505·4 (448·5–566·5)	8·4 (-8·7–22·9)
**2030**	6,248	296 (264–331)	473·5 (422·4–529·3)	14·1 (-1·7–27·5)
**2060**	8,421	387 (350–427)	459·8 (415·8–506·4)	16·7 (2·2–29·0)

Supplementary Table 13: Questionnaire and results of Round 1 of the Delphi Workshop

Original Items	Median	Mean	% agreement	% disagreement
	(IQR)	(COV)	(scores 7,8 & 9)	(scores 1,2,3 & 4)
**Section-1: Conceptual definitions**				
**Item 1: Elimination of transmission:** Interruption of endemic genital wart	**5·0**	**4·7**	**25%**	**39%**
transmission caused by HPV 6 and 11, and limited transmission from imported cases.	**(4·0–5·8)**	**(0·40)**		
**Item-2: Elimination as a public health problem:**	**6·5**	**6·4**	**50%**	**11%**
Transmission of genital warts continues to occur (even in absence of importation) but is	**(5·0–8·0)**	**(0·31)**		
reduced to a level that it does not constitute a public health problem.				
**Section-2: Operational thresholds**				
Short-term control threshold:				
≤4 cases per 10,000 population by year 2030; Equivalent to**:** *A reduction in annual genital*	.. [a]	.. [a]	.. [a]	.. [a]
incidence by 82% by year 2030				
**Item 3:** In your opinion, would the proportion of genital warts caused by non-vaccine	**4·5**	**4·3**	**22%**	**50%**
HPV types have an impact on the control threshold in year 2030? [b]	**(2·2–5·0)**	**(0·52)**		
**Item 4:** In your opinion, would ongoing transmission in Australia due to importation of	**6·0**	**5·6**	**28%**	**11%**
genital warts have an impact on the control threshold in year 2030?	**(5·0–6·7)**	**(0·29)**		
Long-term elimination threshold: *≤1 case per 10,000 population by year 2060;*	.. [a]	.. [a]	.. [a]	.. [a]
Equivalent to: *A reduction in annual genital warts incidence by 95% by year 2060*				
**Item 5:** In your opinion, would the proportion of genital warts caused by non-vaccine	**4·5**	**4·4**	**17%**	**50%**
HPV types have an impact on the elimination threshold in year 2060? [b]	**(3·0–6·0)**	**(0·46)**		
**Item 6:** In your opinion, would ongoing transmission in Australia due to importation of	**6·0**	**5·6**	**33%**	**33%**
genital warts have an impact on the elimination threshold in year 2060?	**(4·0–7·0)**	**(0·40)**		
**Section-3: Intervention coverage**				
**Item 7:** ≥80% coverage of 2-dose girls and boys HPV vaccination program	**7·0**	**7·0**	**56%**	**0%**
	**(6·0–8·0)**	**(0·18)**		
**Section-4: Measuring elimination**				
The 'Bettering the Evaluation and Care of Health' (BEACH) study that used to collect	.. [a]	.. [a]	.. [a]	.. [a]
nationally representative primary care and general practice data in Australia was closed in				
year 2017.				
**Item 8:** In the absence of the BEACH study, should the elimination of genital warts be	**5·0**	**4·8**	**11%**	**39%**
measured via the Genital Warts Surveillance Network (54 sexual health clinics) and	**(3·2–6·0)**	**(0·35)**		
then be adjusted for cases seen at general practice using ASHR-3 estimates of treatment				

ASHR-3: The Australian Study of Health and Relationships 3

Notes:

[a] no quantitative scoring

[b] (item note) -Around 95% of genital warts are caused by HPV 6 and 11. Half of the remaining genital warts are caused by HPV types that are included in qHPV or nonavalent vaccine (example HPV 16, 18, 45, 31 and 52). The remaining 2·5% are caused by non-vaccine HPV types.

Supplementary Information Part 2:

Work undertaken for questionnaire revision as per experts' feedback for the online round of the Delphi process

Section 1 – Conceptual Definition of Elimination:

Item-1: Elimination of transmission – After Delphi workshop, we conducted a review of relevant literature which indicated that discrete geographical variations of HPV 6/11 do not exist, therefore distinguishing between endemic and imported cases through genotyping and sequence analyses is not possible. Ideally, mapping geographically distinct genetic variants for HPV 6/11 would help distinguish endemic from imported transmission. Unfortunately, there is limited literature available on genetic variation of HPV types 6 and 11, and geographical regions do not have unique genetic signatures.[61] An Australian study observed a similar finding where no geographically specific variants for HPV 6 or 11 types were noted.[62] Nonetheless, a study analysing the global genomic diversity of HPV 6 based on 190 complete genome sequences for HPV 6 revealed two variant lineages, A and B and five sub-lineages (B1, B2, B3, B4 and B5). The results showed lineage B is prevalent globally with an association with anogenital infections (notably, sub-lineages B1 and B3), unlike lineage A which predominantly prevails in Asia and was not strongly associated with anogenital infections as compared to lineage B. Therefore, we can conclude that if the majority of anogenital infections (causing warts) are associated with lineage B, markedly, with sub-lineage B1 and B3, and that these are universally prevalent, it would be difficult to distinguish endemic from imported transmission of HPV 6.

Item-2: Elimination as a public health problem - Literature showed that health problems need to meet the following criteria for them to be classified as a public health problem, namely: high burden of disease; large burden in terms of morbidity, quality of life and cost, and feasibility to act on the condition at a community or public health level.[63-65] The prevalence of genital warts in Australia prior to the HPV vaccination program could satisfy the definition of a public health problem as per the aforementioned criteria, namely, high burden of disease (incidence ~22 cases per 10 000 Australians - 74 and 86 per 10 000 population in 25–29-year old men and 20-24-year old women, respectively),[5] large burden in terms of quality of life (psychosocial impact comparable or greater to a diagnosis of CIN 2/3)[66] and cost (over $14 million),[5] and the feasibility to act on the condition at a public health level (via HPV vaccination). Feasibility to act on the condition at a community or public health level: In Australia two HPV vaccines were registered for use in years 2006–07, the bivalent HPV vaccine protecting against high-risk HPV types 16 and 18, and the quadrivalent vaccine which additionally covers the wart-causing types 6 and 11. In 2007, Australia implemented its school-based quadrivalent HPV vaccination program, clearly demonstrating that low-risk HPV infections and the consequent sequelae were part of the overarching health policy. In addition, evidence suggests that due to the additional impact on genital warts, the quadrivalent vaccine is more cost-effective than the bivalent vaccine.[67]

Section 2 - Operational thresholds:

Items-3 and -5: Impact of genital warts caused by non-vaccine HPV types

Some studies showed cross protection from the HPV vaccines to other high-risk non-vaccine HPV types (types 31/33/45);[68,69] however, at the time we calculated these thresholds in early 2018, there was still a gap in the literature to show the same for other non-vaccine HPV types, particularly those associated with the remaining 2·5% genital warts (such as HPV type 39, 40, 48, 51, 55, 59 and 84). In July 2018, a Norwegian study was published analysing the impact of quadrivalent HPV vaccination on vaccine-type and non-vaccine HPV types.[70] They invited three birth cohorts of 17-year-old girls, two vaccine-ineligible cohorts born in 1994 and 1996, and one vaccine-eligible cohort born in 1997 to participate in the study. Approximately 20% of the invited girls participated by delivering a urine sample, that was then tested for HPV infection. Their results showed that prevalence of non-vaccine types was significantly lower among vaccinated cohorts suggesting cross-protection (HPV 33: 53% reduction; HPV 39: 64% reduction; HPV 51: 51% reduction; HPV 59: 48% reduction, any low-risk HPV type, including HPV 40: 47% reduction). A similar impact was seen among unvaccinated girls in the vaccinated cohort suggesting a herd effect.

References:

1. Korostil IA, Ali H, Guy RJ, Donovan B, Law MG, Regan DG. Near elimination of genital warts in Australia predicted with extension of human papillomavirus vaccination to males. *Sex Transm Dis* 2013; **40**: 833–35.
2. Zhang L, Regan DG, Ong JJ, et al. Targeted human papillomavirus vaccination for young men who have sex with men in Australia yields significant population benefits and is cost-effective. *Vaccine* 2017; **35**: 4923–29.
3. Brisson M, Bénard É, Drolet M, et al. Population-level impact, herd immunity, and elimination after human papillomavirus vaccination: a systematic review and meta-analysis of predictions from transmission-dynamic models. *Lancet Public Health* 2016; **1**: e8–e17.
4. Kirby Institute. HIV, viral hepatitis and sexually transmissible infections in Australia: annual surveillance report 2017. 2018. Kirby Institute, UNSW Sydney.
5. Pirotta M, Stein AN, Conway EL, Harrison C, Britt H, Garland SM. Genital warts incidence and healthcare resource utilisation in Australia. *Sex Transm Infect* 2010; **86**: 181–86..
6. National HPV Vaccination Program Register. Coverage Data. 2016. http://www.hpvregister.org.au/research/coverage-data [accessed October 2, 2019].
7. Jin F, Prestage GP, Kippax SC, et al. Risk factors for genital and anal warts in a prospective cohort of HIV-negative homosexual men: the HIM study. *Sex Transm Dis* 2007; **34**: 488–93.
8. Blas MM, Brown B, Menacho L, Alva IE, Silva-Santistebanet A, Carcamo C. HPV prevalence in multiple anatomical sites among men who have sex with men in Peru. *PLoS One* 2015; **10**: e0139524.
9. Manhart LE, Koutsky LA. Do condoms prevent genital HPV infection, external genital warts, or cervical neoplasia?: A meta-analysis. *Sex Transm Dis* 2002; **29**: 725–35.
10. Wen LM, Estcourt C, Simpson J, Mindel A. Risk factors for the acquisition of genital warts: are condoms protective? *Sex Transm Infect* 1999; **75**: 312–16.
11. Australian Bureau of Statistics. 3222.0 - Population Projections, Australia, 2012 (base) to 2101. 2013. http://www.abs.gov.au/ausstats/abs@.nsf/lookup/3222.0Media%20Release12012%20(base)%20to%202101 [accessed October 2, 2019].
12. Tourism Research Australia. International visitor survey results - for year ending Decmeber 2006 to year ending December 2016. 2017. *https://www.tra.gov.au/ArticleDocuments/173/IVS_1_YE_Dec_2016.xlsx.aspx?Embed=Y [accessed October 2, 2019]*.
13. Department of Education and Training Australian Government. International student data 2017; Basic pivot table 2002 onwards. 2017. https://internationaleducation.gov.au/research/International-Student-Data/Pages/InternationalStudentData2017.aspx#Pivot_Table [accessed September 26, 2019].
14. Department of Home Affairs. Department of Immigration and Citizenship Annual Report 2006-07. 2007. https://www.homeaffairs.gov.au/reports-and-pubs/Annualreports/diac-annual-report-2006-07.pdf [accessed October 2, 2019].
15. Renshaw L, Kim J, Fawkes J, Jeffreys E. Migrant sex workers in Australia. *Research and Public Policy no. 131.* Canberra: Australian Institute of Criminology 2015.
16. Callander D. International female sexworks who arrived in Australia within one year of their first visit to a sexual health clinic (based on the data from the Australian Collaboration for Coordinated Enhanced Sentinel Surveillance (ACCESS)) *Unpublished data* 2017.
17. Egan C. Sexual behaviour of international backpackers in the context of travel in Australia. *Doctoral Dessertation* 2009.
18. Department of Education and Training. International student numbers 2014. https://internationaleducation.gov.au/research/Research-Snapshots/Documents/International%20Student%20Numbers%202014.pdf [accessed October 2, 2019].
19. Department of Immigration and Citizenship. Australia's Migration trends 2011-12. 2013. https://www.homeaffairs.gov.au/research-and-stats/files/australian-migration-trends-2011-12.pdf [accessed October 2, 2019].
20. Buckley B, Henschke N, Maayan N, Marshall R, Lutje V, Soares-Weiser K. Anogenital warts: incidence, prevalence, self-reported history and quality of life. Anogenital warts final report. *Cochrane* 2016.
21. Hughes K, Downing J, Bellis MA, Dillion P, Copeland J. The sexual behaviour of British backpackers in Australia. *Sex Transm Infect* 2009; **85**: 477–82.
22. Rosenthal DA, Russell J, Thomson G. The health and wellbeing of international students at an Australian university. *High. Educ.* 2008; **55**: 51–57.
23. Government of United Kingdom. Annual HPV vaccine coverage 2015 to 2016: by local authority and area team. https://www.gov.uk/government/statistics/annual-hpv-vaccine-coverage-2015-to-2016-by-local-authority-and-area-team [accessed October 3, 2019].

24. Poethko-Müller C, Buttmann-Schweiger N. HPV vaccination coverage in German girls: results of the KiGGS study: first follow-up (KiGGS Wave 1). *Bundesgesundheitsblatt, Gesundheitsforschung, Gesundheitsschutz* 2014; **57**: 869–77.
25. Walker TY, Elam-Evans LD, Singleton JA, et al. National, regional, state, and selected local area vaccination coverage among adolescents aged 13–17 years—United States, 2016. *MMWR Morb Mort Wkly Rep* 2017; **66**: 874–82.
26. Uhart M, Adam M, Dahlab A, Bresse X. Loss of chance associated with sub-optimal HPV vaccination coverage rate in France. *Papillomavirus Res* 2017; **3**: 73–79.
27. Ministry of Health New Zealand. HPV immunisation programme; immunisation coverage; 2017. https://www.health.govt.nz/our-work/preventative-health-wellness/immunisation/hpv-immunisation-programme [accessed October 4, 2019].
28. Ingrid Uhnoo. Register-based monitoring of the Swedish HPV vaccination programme. Bergen 2014. In: *Nordic Vaccine Meeting*.
29. ICO Information Centre on HPV and Cancer. Human Papillomavirus and Related Diseases Report; Norway 2017. http://www.hpvcentre.net/statistics/reports/NOR.pdf [accessed October 5, 2019].
30. ICO Information Centre on HPV and Cancer. Human Papillomavirus and Related Diseases Report; Denmark 2017. http://www.hpvcentre.net/statistics/reports/DNK.pdf [accessed October 5, 2019].
31. Bird Y, Obidiya O, Mahmood R, Nwankwo C, Moraros J. Human papillomavirus vaccination uptake in Canada: A systematic review and meta-analysis. *Intl J Prev Med* 2017; **8**:71
32. ICO Information Centre on HPV and Cancer. Human Papillomavirus and Related Diseases Report; Switzerland 2017. http://www.hpvcentre.net/statistics/reports/CHE.pdf [accessed October 5, 2019].
33. Bonanni P, Ferro A, Guerra R, et al. Vaccine coverage in Italy and assessment of the 2012-2014 National Immunization Prevention Plan. *Epidemiol Prev* 2015; **39**: 146–58.
34. ICO Information Centre on HPV and Cancer. Human Papillomavirus and Related Diseases Report; Italy 2017. http://www.hpvcentre.net/statistics/reports/ITA.pdf [accessed October 5, 2019].
35. Buang SN, Jahis R. Lessons Learned: Building The School-based HPV Program in Malaysia and Opportunities for Piggybacking. Bangkok 2016. http://adva.asia/ads/day2/Rohani-Jahis+Saidatul-Norbaya-Buang.pdf [accessed October 5 2019]. In: *Asia Dengue Summit*.
36. Lim SWC, Boo AYY, Tan WC, et al. A cohort survey of the effect of an educational intervention on human papillomavirus vaccine-related knowledge and attitudes among pre-university female students in Singapore. *Proceedings of Singapore Healthcare* 2017; **26**: 5–10.
37. Šubelj M, Učakar V, Kraigher A, Klavs I. Adverse events following school-based vaccination of girls with quadrivalent human papillomavirus vaccine in Slovenia, 2009 to 2013. *Eurosurveillance* 2016; **21**.
38. Limia A, Pachón I. Coverage of human papillomavirus vaccination during the first year of its introduction in Spain. *Eurosurveillance* 2011; **16**: 19873.
39. Feidhmeannacht na Seirbhíse Sláinte, Health Service Executive, Health Protection Surveillance Centre. HPV vaccine uptake in Ireland: 2015/2016. 2016. https://www.hpsc.ie/a-z/vaccinepreventable/vaccination/immunisationuptakestatistics/hpvimmunisationuptakestatistics/File,16039,en.pdf [accessed October 8, 2019].
40. Fisher WA, Laniado H, Shoval H, Hakim M, Bornstein J. Barriers to human papillomavirus vaccine acceptability in Israel. *Vaccine* 2013; **31**: I53–57.
41. ICO Information Centre on HPV and Cancer. Human Papillomavirus and Related Diseases Report; Portugal 2017. http://www.hpvcentre.net/statistics/reports/PRT.pdf [accessed October 6, 2019].
42. Patel H, Wilson E, Vizzotti C, Parston G, Prestt J, Darzi A. Argentina's successful implementation of a national human papillomavirus vaccination program. *Health Aff* 2016; **35**: 301–08.
43. Bachelet VC. Two doses and not three for the human papilloma virus vaccine in Chile. *Medwave* 2016; **16**: e6373
44. ICO Information Centre on HPV and Cancer. Human Papillomavirus and Related Diseases Report; Colombia 2017. http://www.hpvcentre.net/statistics/reports/COL.pdf [accessed October 6, 2019].
45. Public Health Agency of Sweden. Statistics for HPV vaccinations. Secondary Public Health Agency of Sweden. Statistics for HPV vaccinations 2015. https://www.folkhalsomyndigheten.se/folkhalsorapportering-statistik/statistikdatabaser-och-visualisering/vaccinationsstatistik/statistik-for-hpv-vaccinationer/ [accessed September 15, 2019].
46. Department of Home Affairs. Department of Immigration and Citizenship Annual Reports 2006-2015. 2017; https://www.homeaffairs.gov.au/about/reports-publications/reports/annual [accessed September 16, 2019].
47. Domingues CAMAS, Maranhao AGK and Pinto MFS. The introduction of HPV vaccines in Brazil: advances and challenges. *J bras Doenças Sex Transm* 2015; **27**:67-72.

48. National Services Scotland. HPV Immunisation Statistics Scotland. 2017. https://www.isdscotland.org/Health-Topics/Child-Health/Publications/2017-11-28/2017-11-28-HPV-Report.pdf [accessed September 17, 2019].
49. Desai S, Wetten S, Woodhall SC, Peters L, Hughes G, Soldan K. Genital warts and cost of care in England. *Sex Transm Infect* 2011: **87**: 464–8
50. Kraut AA, Schink T, Schulze-Rath R, Mikolajczyk RT, Garbe E. Incidence of anogenital warts in Germany: a population-based cohort study. *BMC Infect Dis* 2010; **10**: 360.
51. Camenga DR, Dunne EF, Desai MM, et al. Incidence of genital warts in adolescents and young adults in an integrated health care delivery system in the United States before human papillomavirus vaccine recommendations. *Sex Transm Dis* 2013; **40**: 534–38.
52. Monsonego J, Breugelmans J, Bouee S, Lafuma A, Benard S, Remy V. Anogenital warts incidence, medical management and costs in women consulting gynaecologists in France. *Gynecol, Obstet Fertil* 2007; **35**: 107–13.
53. Lin C, Lau JT, Ho K-M, Lau M-C, Tsui H-Y, Lo K-K. Incidence of genital warts among the Hong Kong general adult population. *BMC Infect. Dis* 2010; **10**: 272.
54. van den Broek IV, Verheij RA, van Dijk CE, Koedijk FD, van der Sande MA, van Bergen JE. Trends in sexually transmitted infections in the Netherlands, combining surveillance data from general practices and sexually transmitted infection centers. *BMC Fam Pract* 2010; **11**: 39.
55. Sasagawa T, Ito F, Ideta K, Irie T. Estimation of the incidence of HPV related diseases in Japan (J-Hers). Berlin 2011. In: *the 27th International Papillomavirus Conference and Clinical Workshop.*
56. Hsueh P-R. Human papillomavirus, genital warts, and vaccines. *J Microbiol Immunol Infect* 2009; **42**: 101–06.
57. Cho C-Y, Lo Y-C, Hung M-C, Lai CC, Chen CJ, Wu KG. Risk of cancer in patients with genital warts: A nationwide, population-based cohort study in Taiwan. *PloS one* 2017; **12**: e0183183.
58. Leaungwutiwong P, Bamrungsak B, Jittmittraphap A, et al. Molecular genotyping of human papillomavirus l1 gene in low-risk and high-risk populations in Bangkok. *Sex Transm Dis* 2015; **42**: 208–17.
59. Su S, Chow EP, Muessig KE, et al. Sustained high prevalence of viral hepatitis and sexually transmissible infections among female sex workers in China: a systematic review and meta-analysis. *BMC Infect Dis* 2015; **16**: 2.
60. O'Connor C, Berry G, Rohrsheim R, Donovan B. Sexual health and use of condoms among local and international sex workers in Sydney. *Sex Transm Infect* 1996; **72**: 47–51.
61. Heinzel PA, Chan S-Y, Ho L, et al. Variation of human papillomavirus type 6 (HPV-6) and HPV-11 genomes sampled throughout the world. *J Clin Microbiol* 1995; **33**: 1746–54..
62. Danielewski JA, Garland SM, McCloskey J, Hillman RJ, Tabrizi, SN. Human papillomavirus type 6 and 11 genetic variants found in 71 oral and anogenital epithelial samples from Australia. *PloS one* 2013; **8**: e63892.
63. Saaddine JB, Narayan KV and Vinicor F. Vision loss: a public health problem? *Ophthalmology* 2003; **110**: 253–54.
64. Vinicor F. Is diabetes a public-health disorder? *Diabetes care* 1994; **17**: 22–27.
65. Schoolwerth AC, Engelgau MM, Rufo KH, et al. Chronic Kidney Disease: A Public Health Problem That Needs a Public Health Action Plan. *Prev Chronic Dis* 2006; **3**: A57.
66. Pirotta M, Ung L, Stein A, et al. The psychosocial burden of human papillomavirus related disease and screening interventions. *Sex Transm Infect* 2009; **85**: 508–13.
67. Damm O, Horn J, Mikolajczyk RT, et al. Cost-effectiveness of human papillomavirus vaccination in Germany. *Cost Eff Resour Alloc* 2017; **15**: 18.
68. Brown DR, Kjaer SK, Sigurdsson K, et al. The impact of quadrivalent human papillomavirus (HPV; types 6, 11, 16, and 18) L1 virus-like particle vaccine on infection and disease due to oncogenic nonvaccine HPV types in generally HPV-naive women aged 16–26 years. *J Infect Dis* 2009; **199**: 926–35.
69. Wheeler CM, Kjaer SK, Sigurdsson K, et al. The impact of quadrivalent human papillomavirus (HPV; types 6, 11, 16, and 18) L1 virus-like particle vaccine on infection and disease due to oncogenic nonvaccine HPV types in sexually active women aged 16–26 years. *J Infect Dis* 2009; **199**: 936–44.
70. Feiring B, Laake I, Christiansen IK, et al. Substantial decline in prevalence of vaccine-type and nonvaccine-type human papillomavirus (HPV) in vaccinated and unvaccinated girls 5 years after implementing HPV vaccine in Norway. *J Infect Dis* 2018; **218**: 1900–10.
